# Supplementary material for: General heterostructure strategy of photothermal materials for scalable solar-heating hydrogen production without the consumption of artificial energy
Source: Nat Commun. 2022 Feb 9;13:776. doi: 10.1038/s41467-022-28364-y (PMC8828830; doi:10.1038/s41467-022-28364-y)
Supplement: Supplementary file 1 — Supplementary Information [file 41467_2022_28364_MOESM1_ESM.pdf]

“Supplementary Information for [General Heterostructure Strategy of Photothermal Materials for Scalable Solar-heating Hydrogen Production Without the Consumption of Artificial Energy]”

Yaguang Li et al.

Methods

The deposition of Ti_2O_3, Cu_2Se, Cu_2S films

The synthesis of Ti_2O_3/Cu, Cu_2Se/Cu, Cu_2S/Cu shown in Supplementary Fig. 4a, Supplementary Fig. 4b, Supplementary Fig. 4c, Supplementary Fig. 6a, Supplementary Fig. 6b, Supplementary Fig. 6c, Supplementary Fig. 6d, Supplementary Fig. 6e, Supplementary Fig. 6f was similar to that of Bi_2Te_3/Cu with 100 nm thickness of Bi_2Te_3. And the only difference was changing the Bi_2Te_3 target by Ti_2O_3 target, Cu_2Se target or Cu_2S target. The sputtering time of films shown in Supplementary Fig. 4a, Supplementary Fig. 4b, Supplementary Fig. 4c, Supplementary Fig. 6a, Supplementary Fig. 6b, Supplementary Fig. 6c, Supplementary Fig. 6d, Supplementary Fig. 6e, Supplementary Fig. 6f was 6 min, 6 min, 6 min, 1 min, 1 min, 1 min, 3 min, 3 min, 3 min, respectively.

For the films shown in Supplementary Fig. 6g, Supplementary Fig. 6h, Supplementary Fig. 6i, the sputtering pressure was 5×10^{-1} Pa and the sputtering time was 10 min.

Characterizations

The overall composition of the prepared samples were studied by the powder X-ray diffraction (XRD), which was performed on a Bede D1 system operated at 20 kV and 30 mA with Cu Kα radiation (λ= 1.5406 Å). The scanning electron microscopy (SEM) images were tested with the FEI Nova NanoSEM450 (Czech Republic). Transmission electron microscopy (TEM, ARM 200 F and JEOL F200+) was used to identify the morphology and the crystal structure of the nanostructures. Zennium_Pro (Zahner, Germany) was an electrochemical workstation. The ultraviolet-visible-infrared absorption spectrum (UV-vis-IR) and IR emission were tested by Hitachi Limited U4100 (Japan) and FTIR spectrometer (Bruker,

VERTEX 70 FT-IR). IR photographs were taken with a camera (Fluke Ti300, America). N_2-sorption isotherms were collected on a Belsorb-Max system. Brunauer-Emmett-Teller (BET) specific surface areas were calculated from adsorption data. The thickness of catalysts was tested by AFM (MFP-3D Origin+, Oxford Instruments). The IR emissivity was tested by FTIR equipped with an integrating sphere. And the IR emissivity of pure Bi_2Te_3 film, Bi_2Te_3 film/Cu with 3 μm, 100 nm, 15 nm thicknesses of Bi_2Te_3, pure Cu film is 0.91, 0.6, 0.05, 0.04, 0.03, respectively. The IR radiation of samples was tested by integrating sphere equipped FTIR with a heater substrate.

Thermalcatalytic MSR

The thermalcatalytic activity of hydrogen generation from MSR was tested by the fixed-bed reactor (XM190708-007, DALIAN ZHONGJIARUILIN LIQUID TECHNOLOGY CO., LTD) in continuous flow form. Typically, 10 mg of catalyst was placed in a quartz flow reactor. The feed gas of 50 sccm Ar was regulated by the mass flow controller for MSR with 0.1 sccm of feed methanol+water (the volume ratio of methanol to water is 1:1.3). The reaction products were tested by gas chromatograph (GC) 7890A equipped with FID and TCD detectors.

Solar-heating MSR

The solar-heating thermalcatalytic MSR was tested as follows: 10 g of catalysts were loaded in Bi_2Te_3/Cu-based device, and irradiated by a xenon lamp (HP-2-4000). In this test, methanol and water were mixed as a solution with the volume ratio of methanol and water = 1:1.6, and the mixed solution was then pumped into the system. We first removed CO_2 in the produced gas through NaOH solution (5 M), and a flowmeter (MV-192-H2, Bronkhorst) was used to measure the flow rate, which was recognized as the rate of hydrogen. The outlet gases were tested by gas chromatograph (GC) 7890A equipped with FID and TCD detectors. The ambient temperature was constant at 30 °C.

The hydrogen generation rate for per gram of catalyst (δ, mmol g^{-1} h^{-1}) from sunlight driven thermal catalysis system was calculated as follows:

δ (mmol g^{-1} h^{-1}) = $(1000*L/24.5)/G$ (1)

L was the gas flow rate (L h^{-1}), G was the weight of catalysts (10 g).

Outdoor solar-heating MSR

The scalable system was made up of the Bi_2Te_3/Cu-based device with 6 m of length and 42 mm of diameter and a parabolic reflector with 6 m of length and 1 m of width. The install of the parabolic reflector was based on the ecliptic plane to make sunlight focus on the light selective absorbing device. 7.4 kg of CuZnAl NS was loaded in the Bi_2Te_3/Cu-based device and outdoor sunlight was used as the light source. In the outdoor test, methanol and water were mixed as a solution with the volume ratio of methanol and water = 1:1.6, and the mixed solution was then pumped into this system. To analyze gas product hydrogen, we first removed CO_2 in the produced gas through NaOH solution (5 M), and a vortex flowmeter was used to measure the instantaneous and cumulative flow of the treated gas (the flow rate of the flowmeter was calibrated according to pure hydrogen), which were defaulted as the data of hydrogen flow. The outdoor sunlight driven thermal MSR reactions were operated from 8:00 to 17:00 on April 08, 2021, in Baoding, China.

Supplementary Table 1 The solar absorptivity, IR emissivity, 1 Sun irradiated temperature of Bi_2Te_3/Cu film with different thickness of Bi_2Te_3. A CaF_2 glass fully covered each sample, and the vacuum degree of this equipment was 1.0×10^{-3} Pa.

Thickness	Solar absorptivity	IR emissivity	1 Sun irradiated temperature
15 nm	43 %	4 %	172 °C
100 nm	89 %	5 %	317 °C
3 μm	94 %	60 %	97 °C

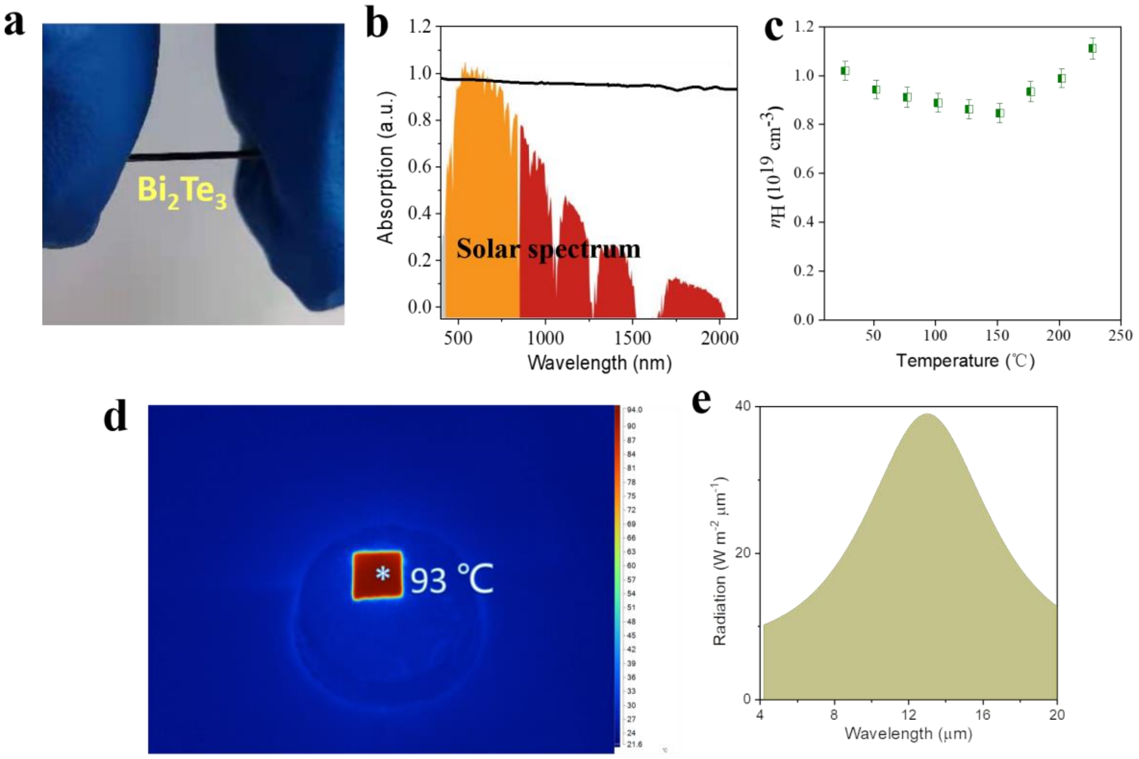

Supplementary Fig. 1 a, b Photograph and normalized light absorption spectra of pure Bi_2Te_3 film. **c** The carrier concentration of pure Bi_2Te_3 film under different temperature. **d** IR image of vacuum protected pure Bi_2Te_3 film under 1 Sun irradiation. A CaF_2 glass fully covered the samples, and the vacuum degree of this equipment was 1.0×10^{-3} Pa. **e** The IR radiation ranging from 4 μm to 20 μm of the pure Bi_2Te_3 film at 93 °C.

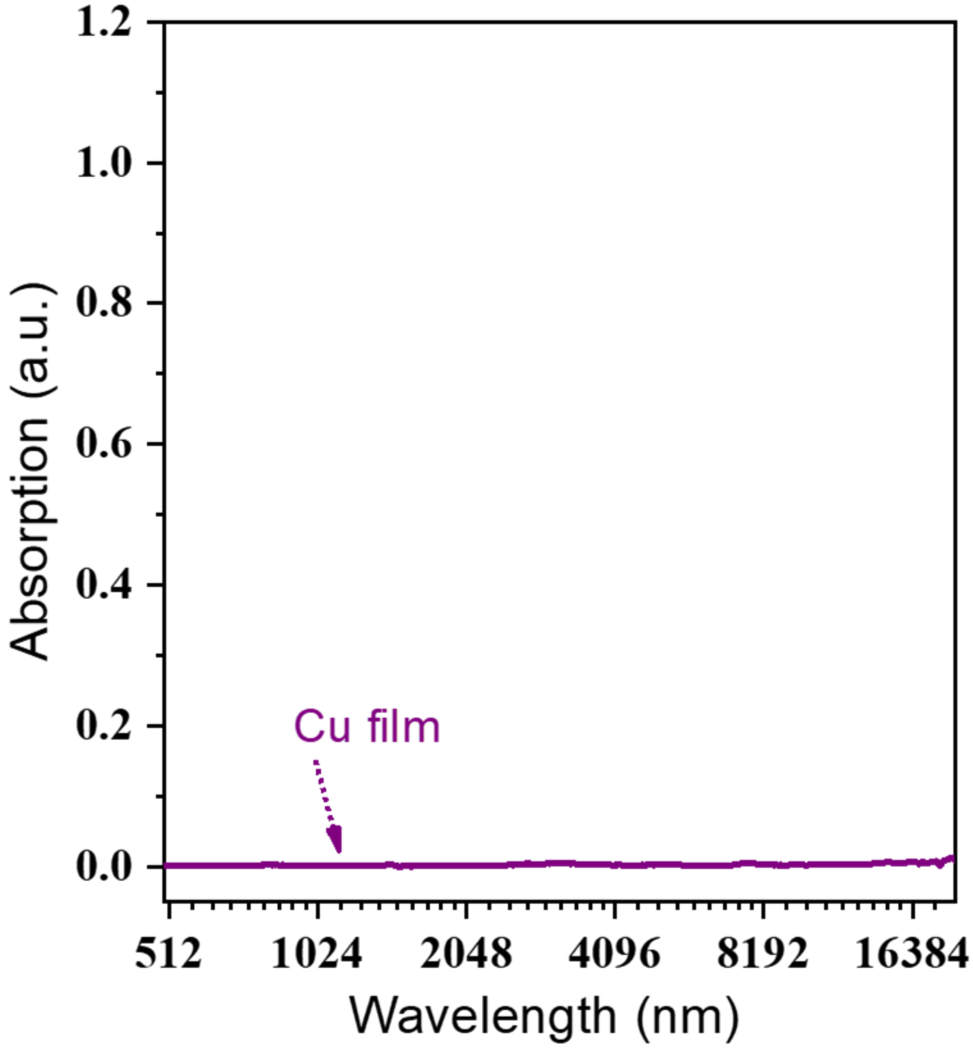

Supplementary Fig. 2 The normalized light absorption spectrum of polished Cu film.

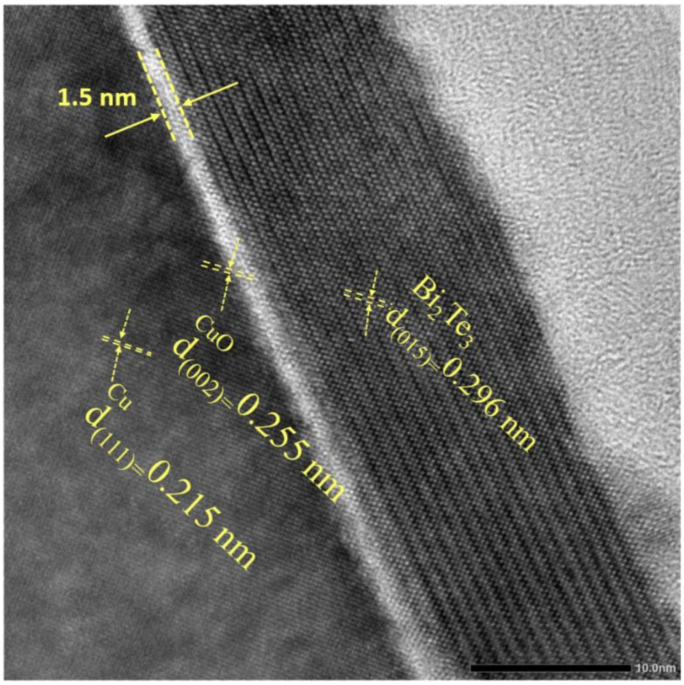

Supplementary Fig. 3 The lattice fringes of the Bi_2Te_3/Cu film.

Supplementary Fig. 3 showed the crystal planes of Cu (111), CuO (002), and Bi_2Te_3 (015) grown in the interface. This interface layer of CuO may be caused by the intrinsic oxidation of the Cu substrate in air. Moreover, the CuO interface layer is ~ 1.5 nm thick, which is so thin that the sunlight converted heat energy in the Bi_2Te_3 layer can be smoothly transferred to the Cu layer, thus heating the device and catalyst efficiently.

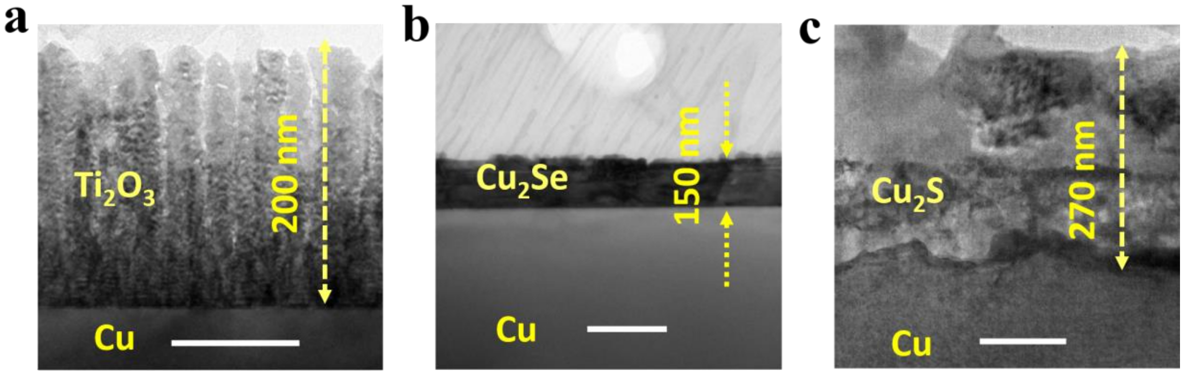

Supplementary Fig. 4 a, b, c TEM images of Ti_2O_3/Cu, Cu_2Se/Cu, Cu_2S/Cu heterostructures. The scale bars in **a**, **b**, **c** are 100, 200, 100 nm, respectively.

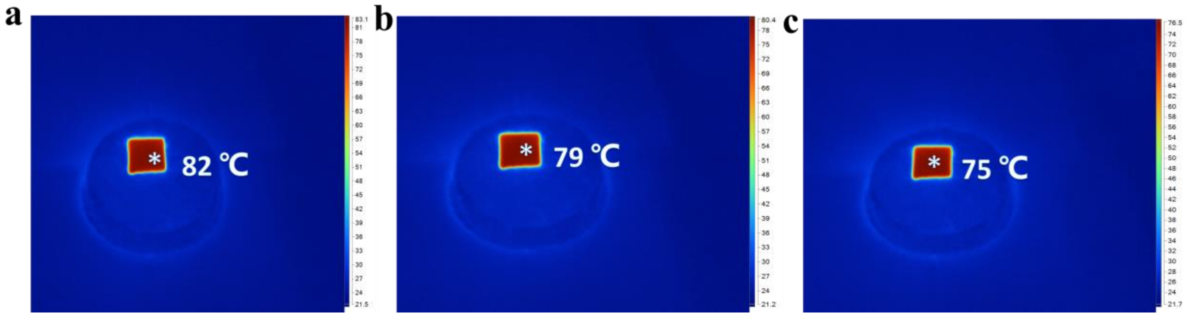

Supplementary Fig. 5 The IR images of vacuum protected Ti_2O_3 **a**, Cu_2Se **b** and Cu_2S **c**, under 1 Sun irradiation. A CaF_2 glass fully covered the materials, and the vacuum degree of this equipment was 1.0×10^{-3} Pa.

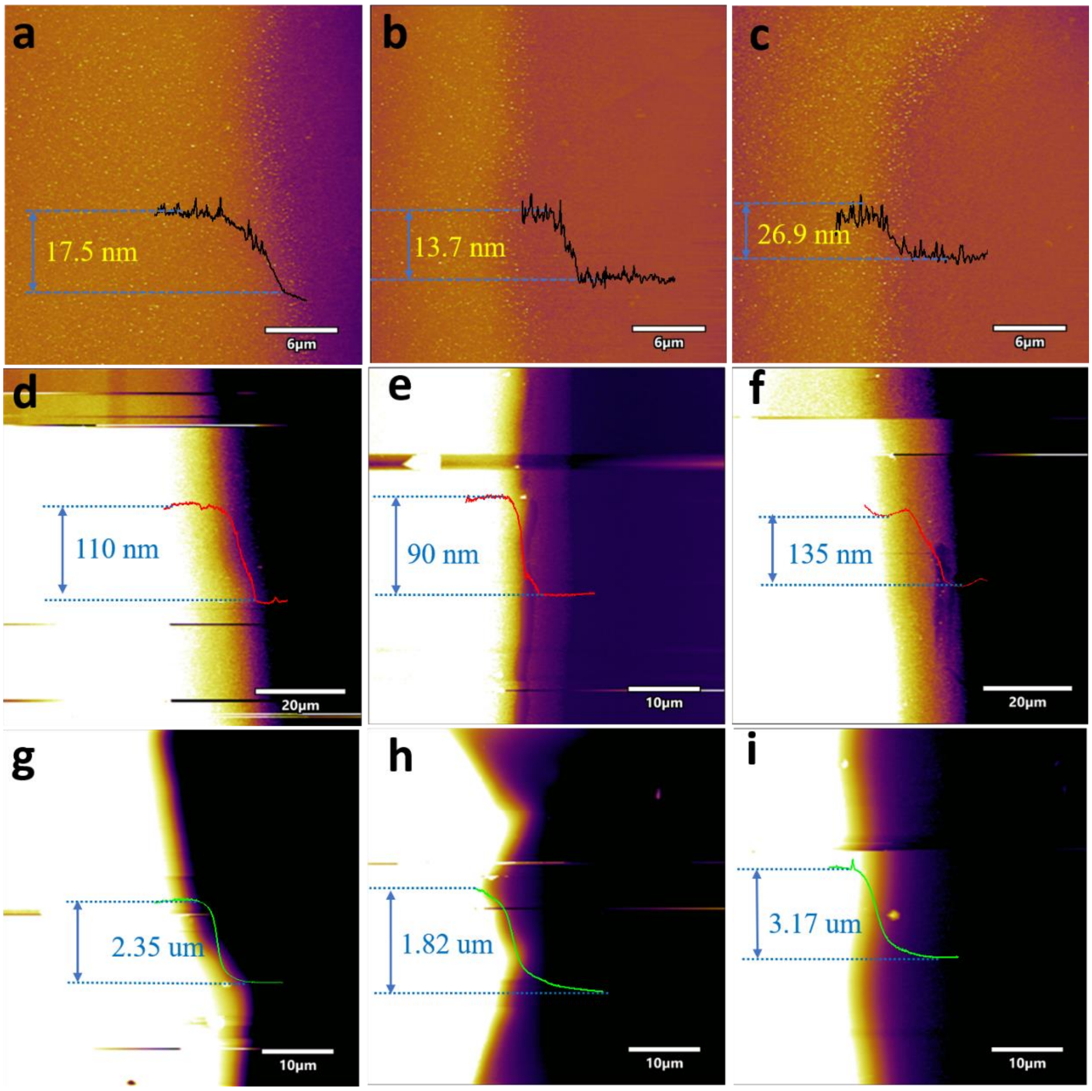

Supplementary Fig. 6 AFM images of **a** Ti_2O_3/Cu with 17.5 nm thickness of Ti_2O_3, **b** Cu_2Se/Cu with 13.7 nm thickness of Cu_2Se, **c** Cu_2S/Cu with 26.9 nm thickness of Cu_2S, **d** Ti_2O_3/Cu with 110 nm thickness of Ti_2O_3, **e** Cu_2Se/Cu with 90 nm thickness of Cu_2Se, **f** Cu_2S/Cu with 135 nm thickness of Cu_2S, **g** Ti_2O_3/Cu with 2.35 μm thickness of Ti_2O_3, **h** Cu_2Se/Cu with 1.82 μm thickness of Cu_2Se, **i** Cu_2S/Cu with 3.17 μm thickness of Cu_2S.

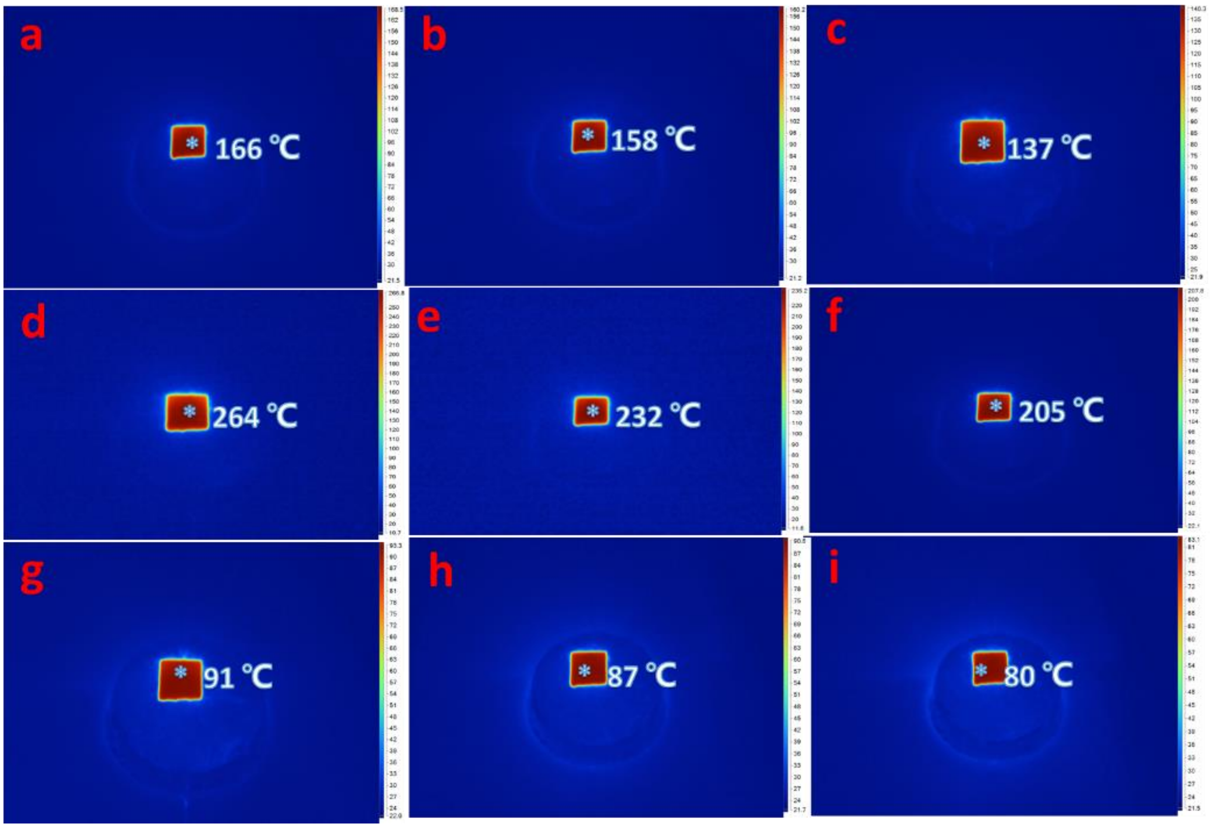

Supplementary Fig. 7 The corresponding IR images of vacuum protected **a** Ti_2O_3/Cu with 17.5 nm thickness of Ti_2O_3, **b** Cu_2Se/Cu with 13.7 nm thickness of Cu_2Se, **c** Cu_2S/Cu with 26.9 nm thickness of Cu_2S, **d** Ti_2O_3/Cu with 110 nm thickness of Ti_2O_3, **e** Cu_2Se/Cu with 90 nm thickness of Cu_2Se, **f** Cu_2S/Cu with 135 nm thickness of Cu_2S, **g** Ti_2O_3/Cu with 2.35 μm thickness of Ti_2O_3, **h** Cu_2Se/Cu with 1.82 μm thickness of Cu_2Se, **i** Cu_2S/Cu with 3.17 μm thickness of Cu_2S, under 1 Sun irradiation. A CaF_2 glass fully covered the materials, and the vacuum degree of this equipment was 1.0×10^{-3} Pa.

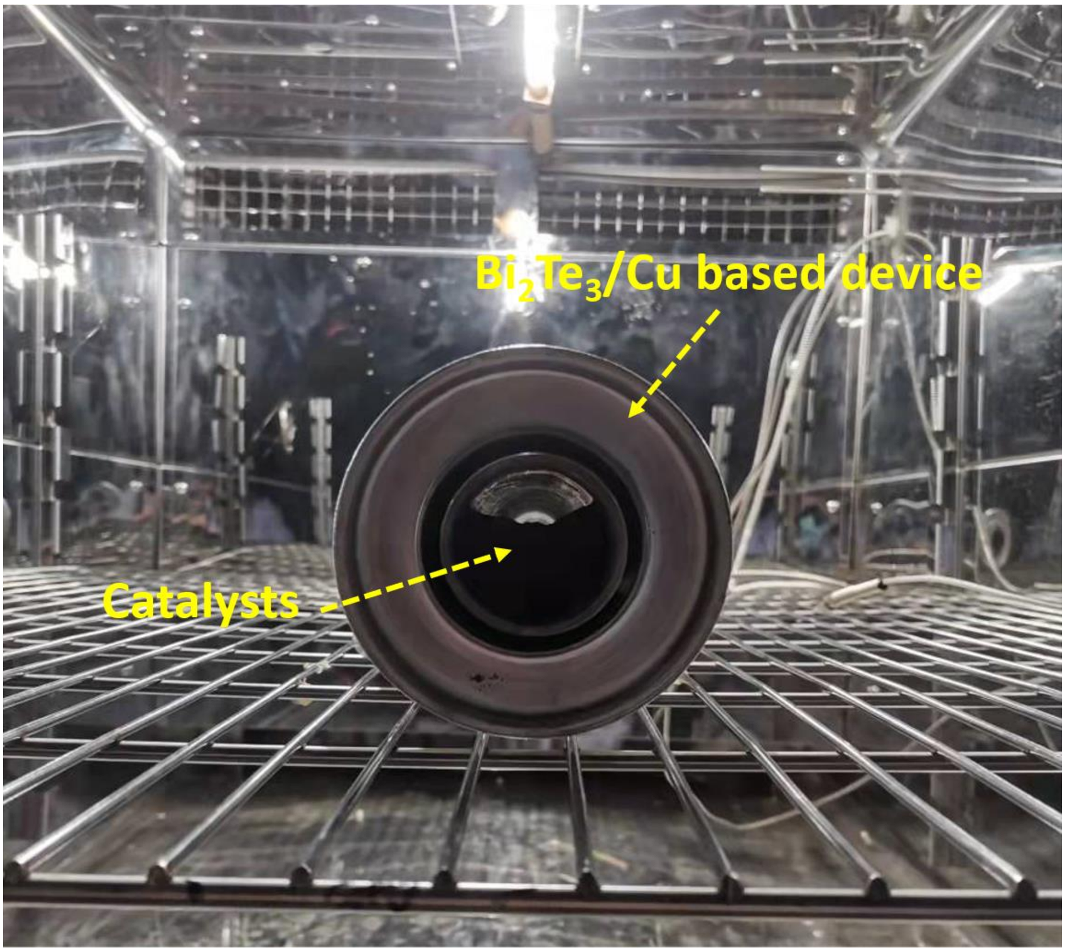

Supplementary Fig. 8 The picture of catalysts loaded in Bi_2Te_3/Cu-based device.

When sunlight directly illuminated the solar heating device, the layer of Bi_2Te_3 could efficiently convert solar energy to thermal energy and radiate little infrared light; the inner layer of Cu could stop infrared emission of catalysts; while the outer vacuum layer can block the thermal conduction from the inner device. In this way, we could create a high temperature to drive MSR under solar illumination (Supplementary Fig. 8).

The key physics of this hybrid is that the infrared radiation is directly proportional to the amount of sunlight absorber (Bi_2Te_3). Therefore, the thinner the thickness of Bi_2Te_3, the less its heat radiation will be. At the same time, if the thickness of Bi_2Te_3 film is less than 100 nm, its ability to absorb sunlight will be greatly reduced. Therefore, controlling the thickness of Bi_2Te_3 to about 100 nm can achieve the balance of high sunlight absorption and low infrared radiation to produce high sunlight irradiation temperature.

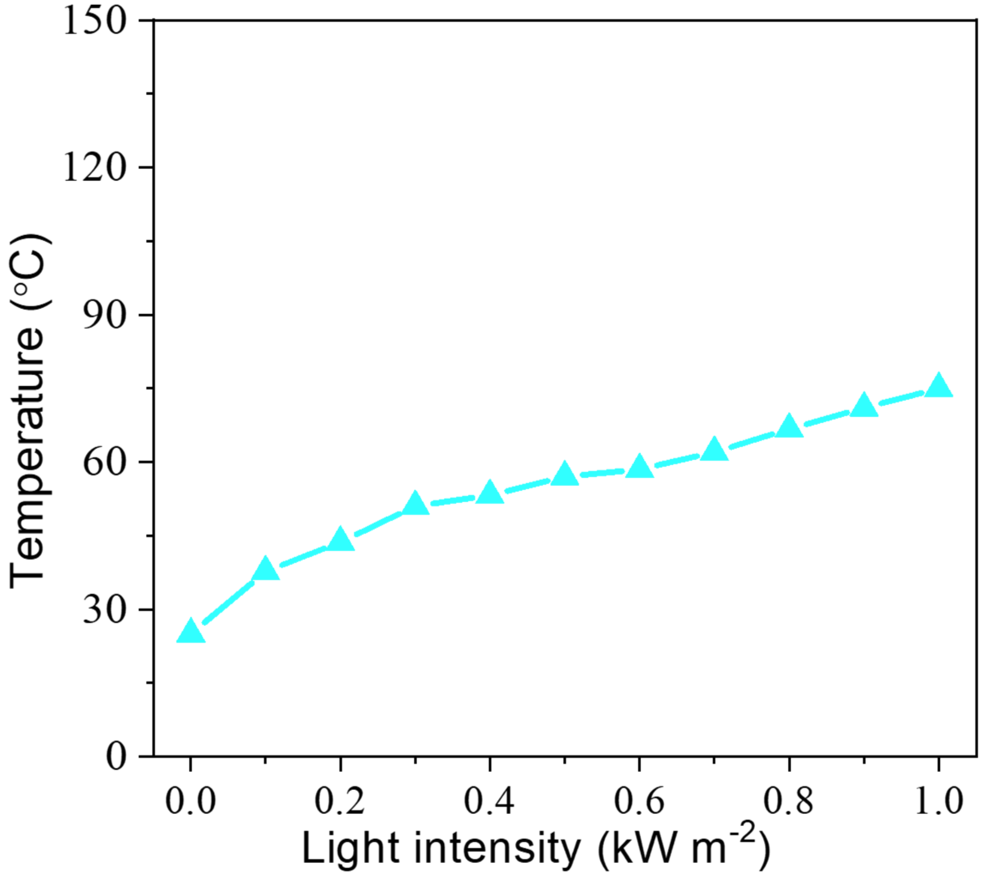

Supplementary Fig. 9 The temperature of the CuZnAl catalyst loaded in a quartz tube, under different intensities of solar irradiation.

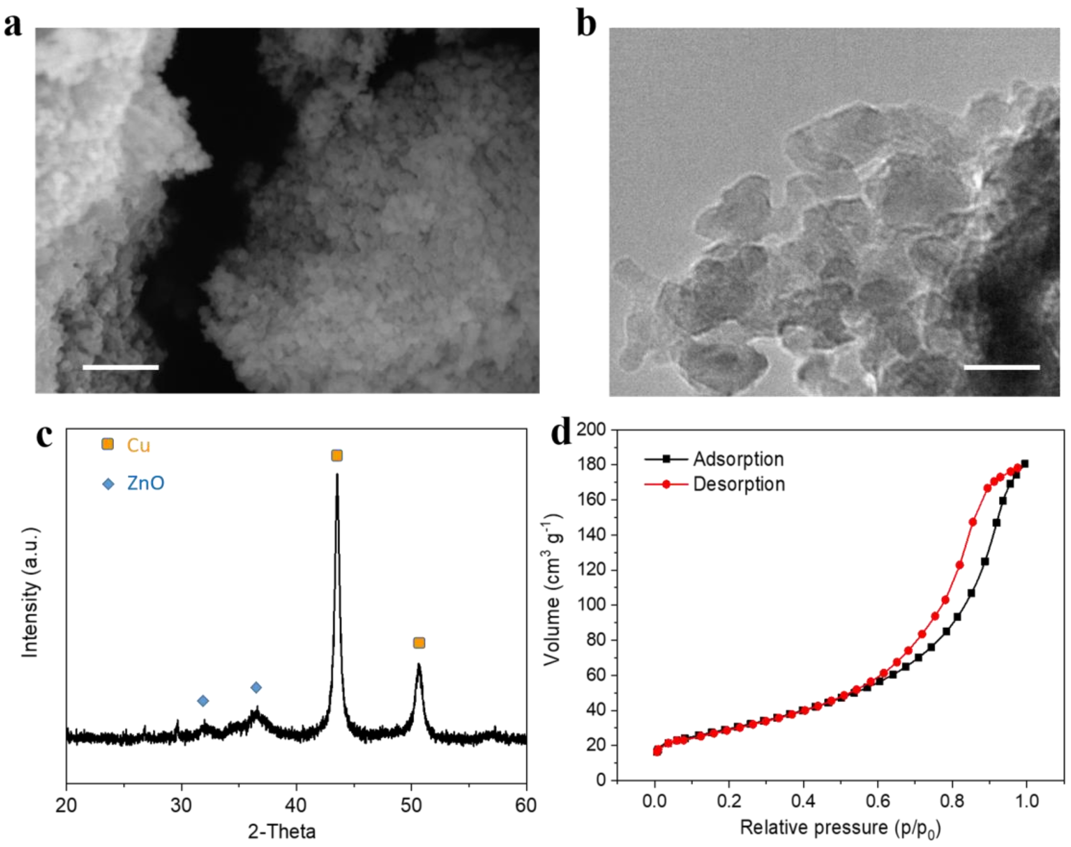

Supplementary Fig. 10 SEM image **a**, TEM image **b**, XRD pattern **c**, nitrogen adsorption and desorption isotherm **d** of commercial CuZnAl. The scale bars in **a**, **b** are 200, 20 nm, respectively.

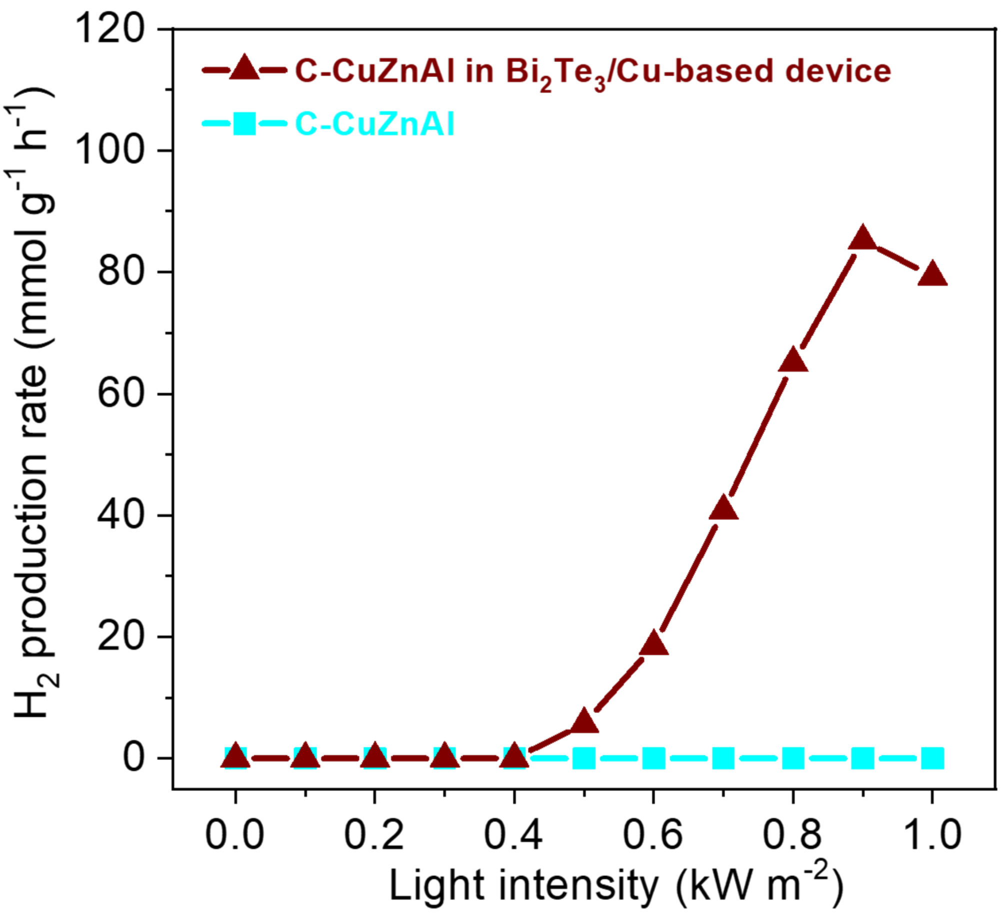

Supplementary Fig. 11 The hydrogen production rates from two MSRs of C-CuZnAl loaded in the Bi_2Te_3/Cu-based device and C-CuZnAl not in the device but directly under different sunlight irradiations.

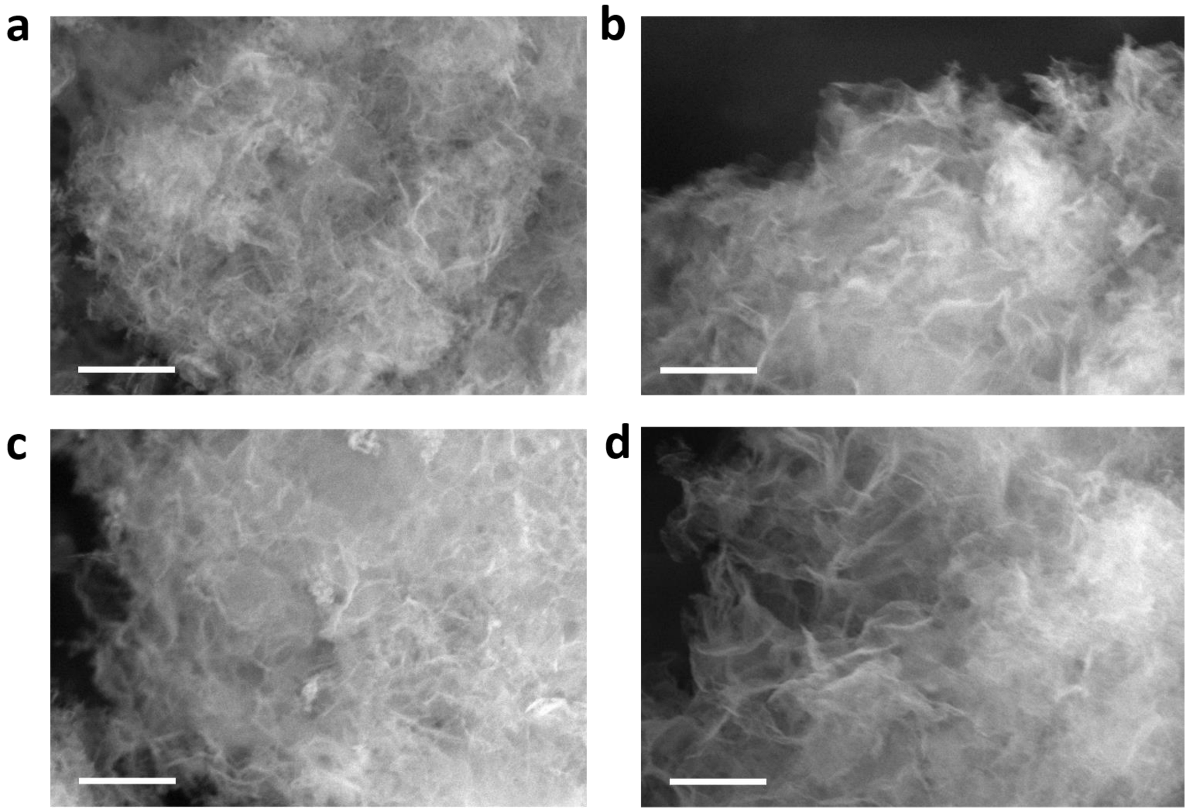

Supplementary Fig. 12 SEM images of CuZnAl NS with PVP/CuZnAl precursor ratio of 2 **a**, 4 **b**, 6 **c**, 8 **d**. The Cu/Zn/Al ratio is 6/3/1. The scale bars in **a**, **b**, **c, d** are 200, 300, 500, 500 nm, respectively.

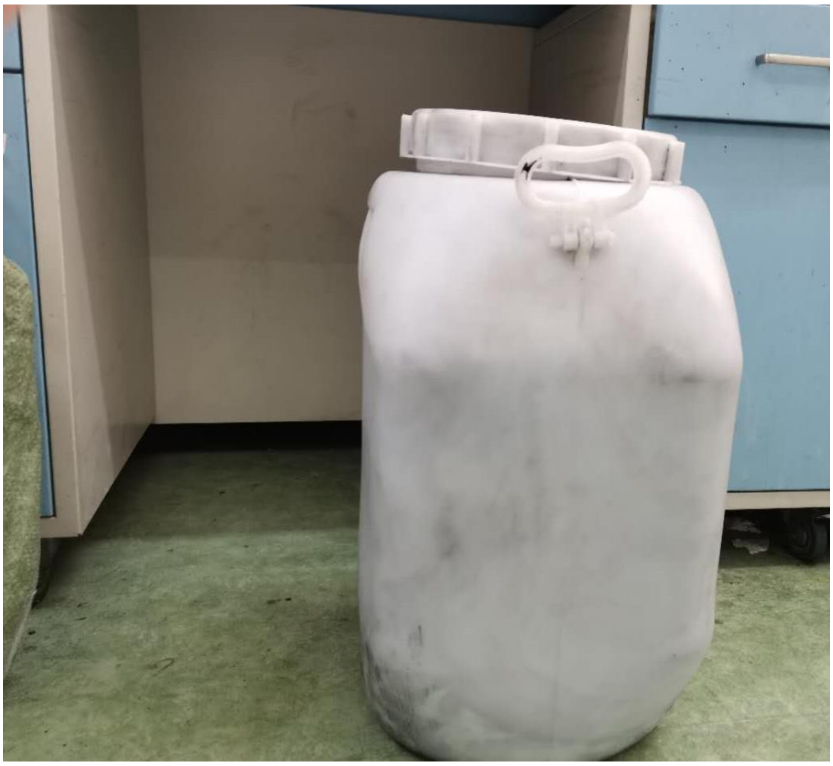

Supplementary Fig. 13 Photograph of the bottle full of as-synthesized CuZnAl NS.

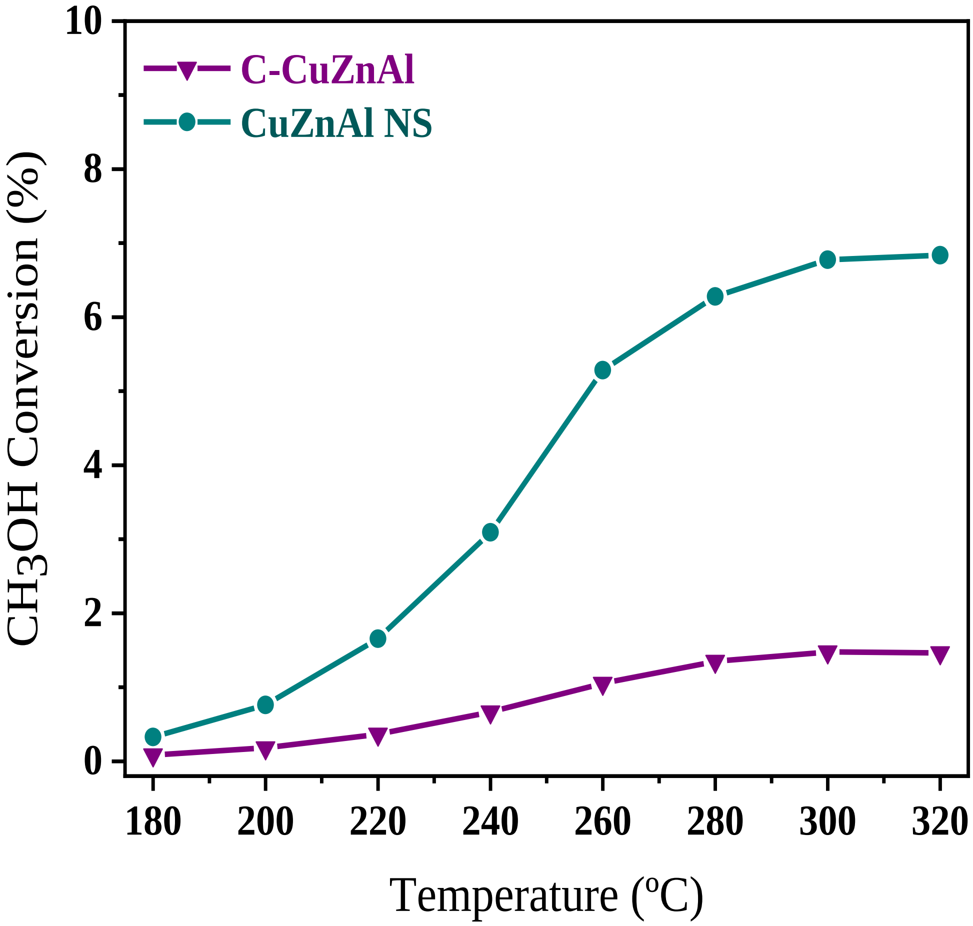

Supplementary Fig. 14 Methanol conversion from MSR of CuZnAl NS and C-CuZnAl at different temperatures.

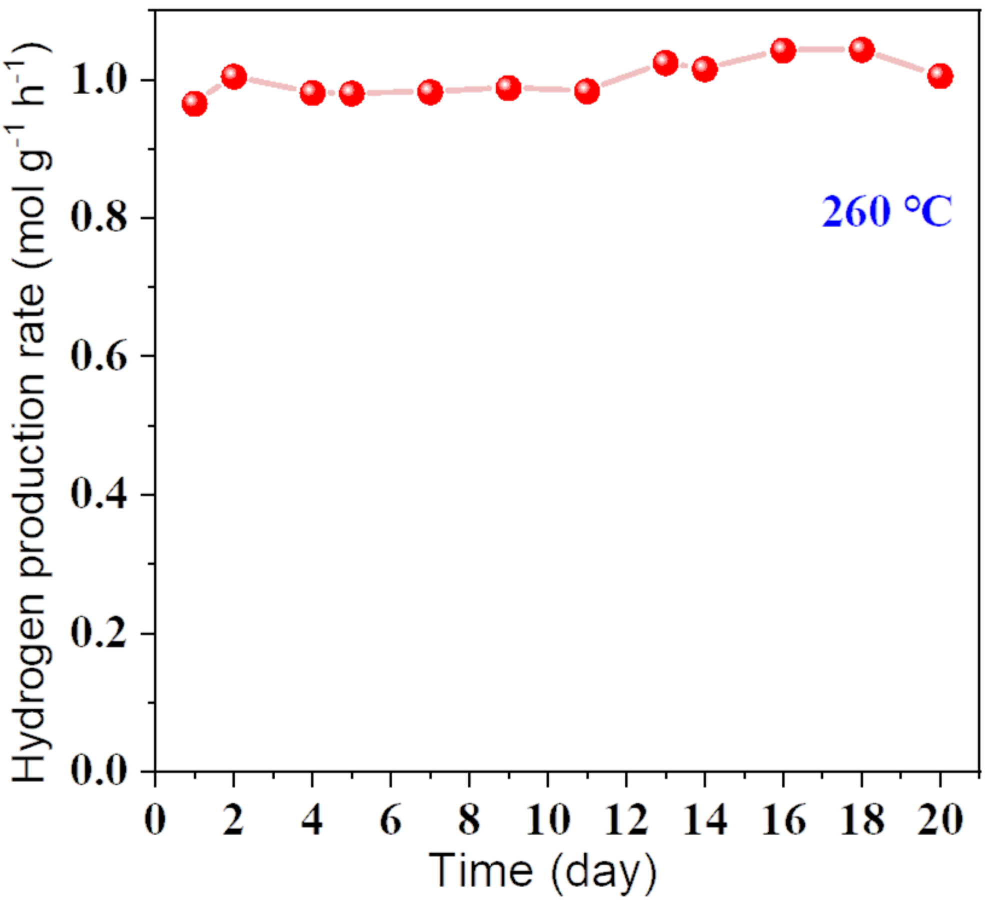

Supplementary Fig. 15 The MSR stability of CuZnAl NS at 260 °C. Test condition: 10 mg of catalyst, 50 sccm of Ar, 0.1 sccm of methanol (the volume ratio of methanol to water=1:1.3).

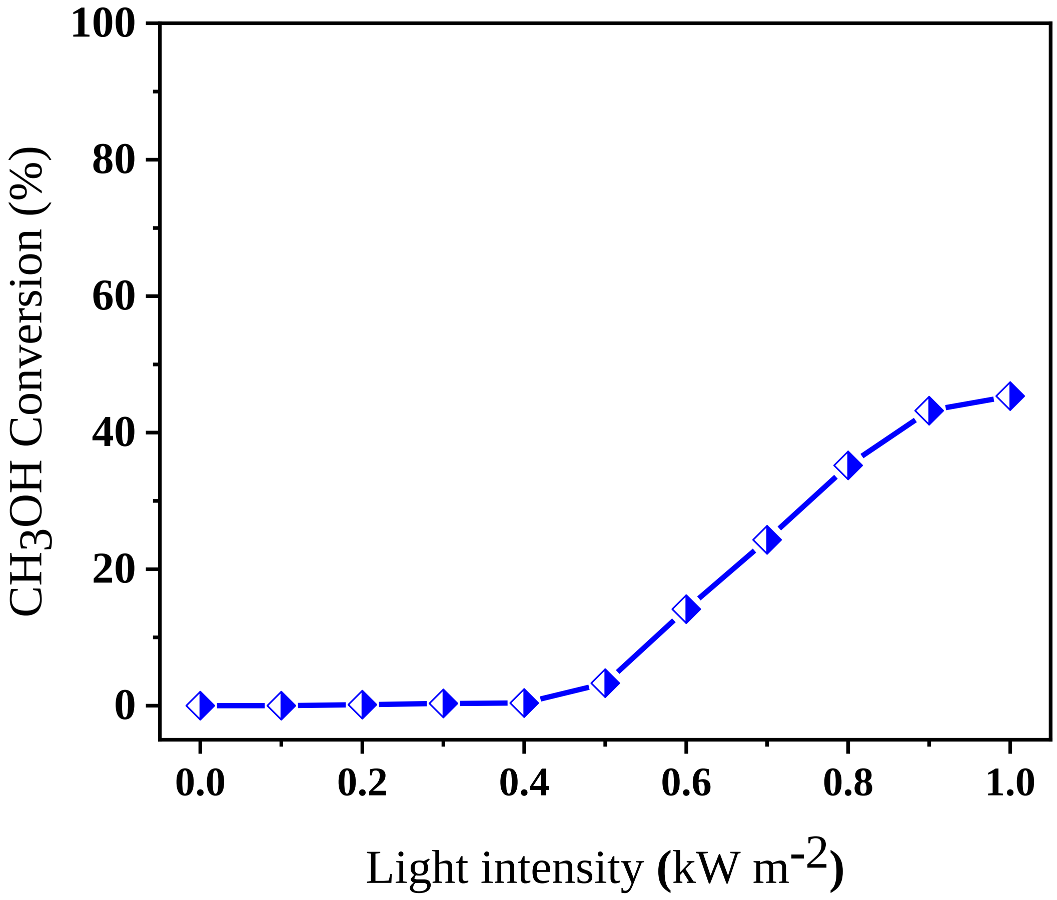

Supplementary Fig. 16 Methanol conversion of CuZnAl NS loaded in the Bi_2Te_3/Cu-based device under different sunlight irradiations.

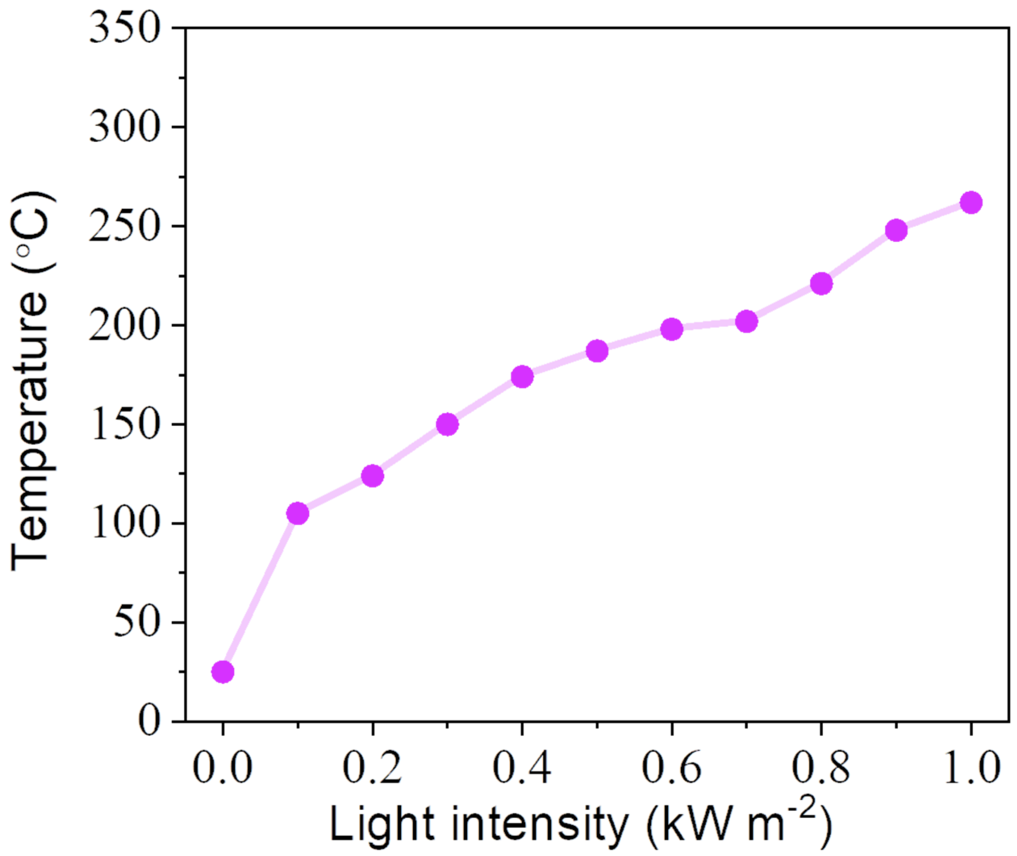

Supplementary Fig. 17 The sunlight driven temperature of the CuZnAl catalyst loaded in the Bi_2Te_3/Cu-based device during MSR.

During the solar heating MSR, the temperature of CuZnAl catalyst loaded in the Bi_2Te_3/Cu-based device was 262, 248, 221, 202, 198, 187, 174, 150, 124, 105 °C under 1, 0.9, 0.8, 0.7, 0.6, 0.5, 0.4, 0.3, 0.2, 0.1 Sun irradaition, respectively.

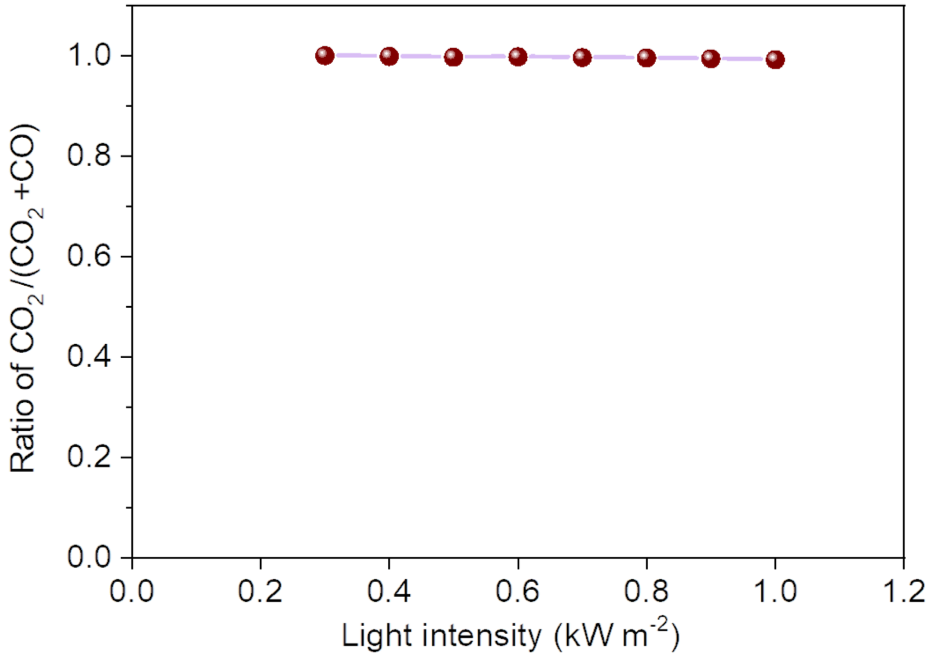

Supplementary Fig. 18 CO_2 selectivity of CuZnAl NS in the Bi_2Te_3/Cu-based device under different sunlight irradiations.

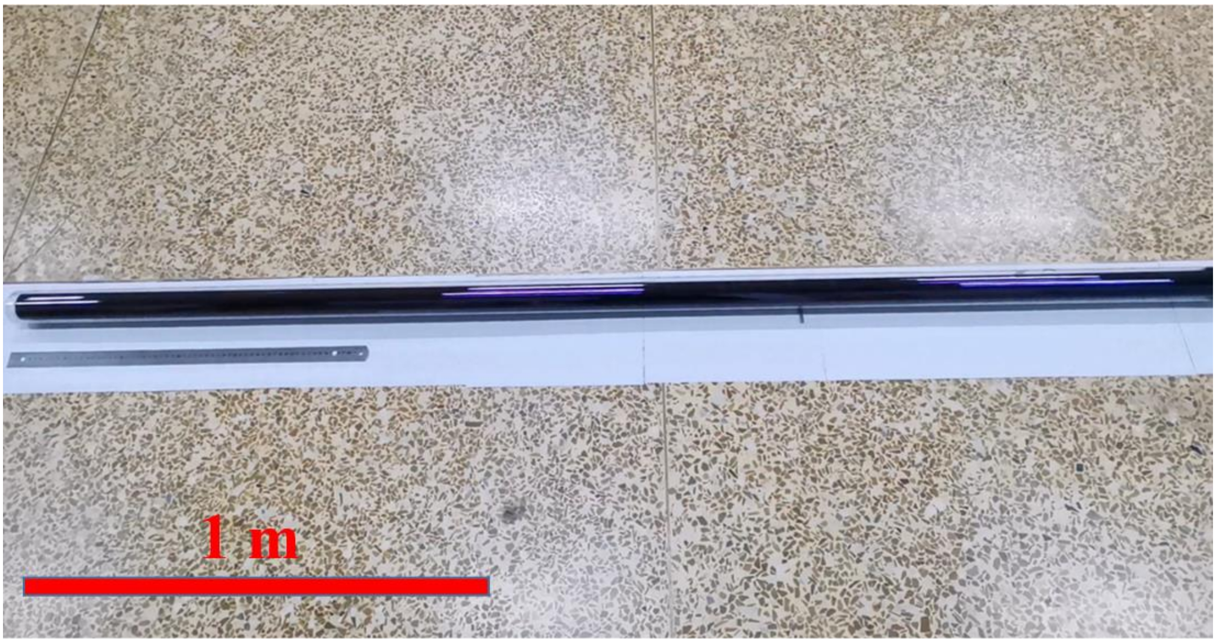

Supplementary Fig. 19 The photograph of a Bi_2Te_3/Cu-based device with ≥2 m length.

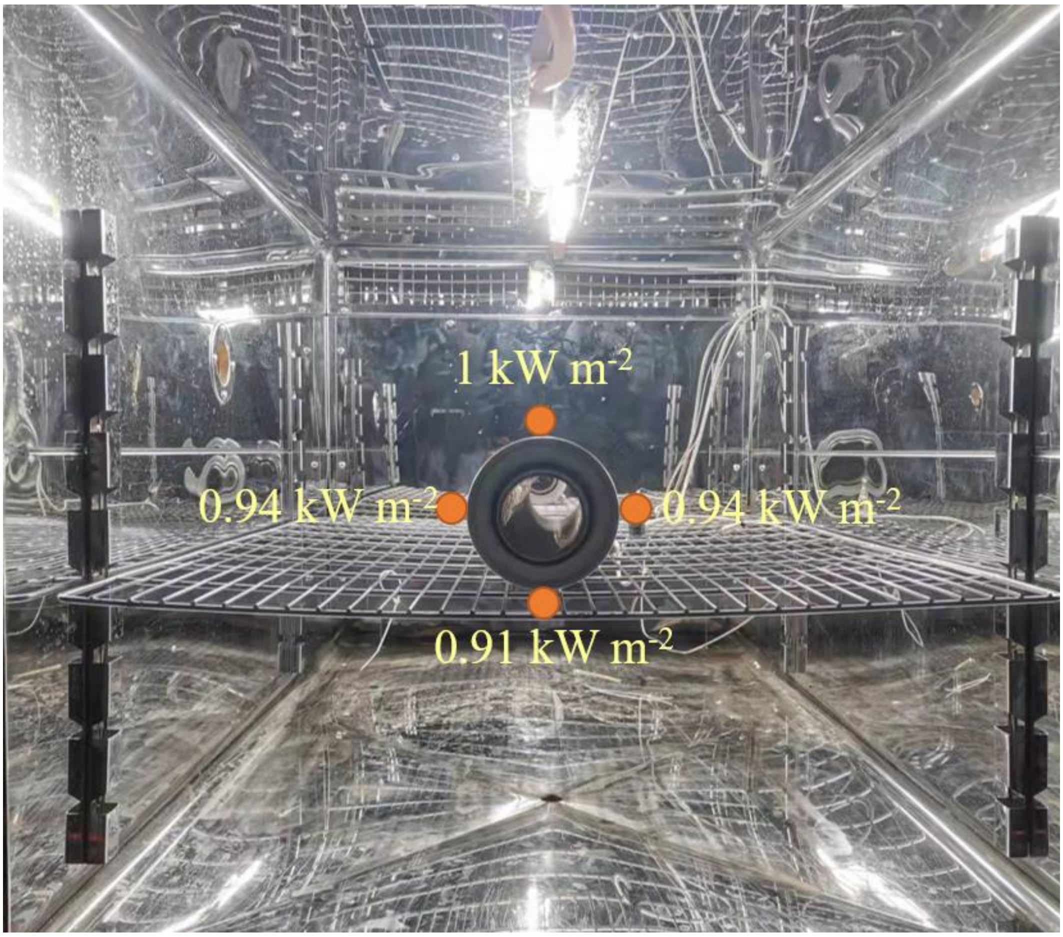

Supplementary Fig. 20 The light irradiation condition of Bi_2Te_3/Cu-based device.

As shown in Fig. 3b, the Bi_2Te_3/Cu layer was full covered on the surface of reaction tube. We showed the photo of Bi_2Te_3/Cu-based device under simulated 1 Sun light source irradiation in Supplementary Fig. 20. Although the light source irradiated the device directly from the top, the side and bottom of the device can still be irradiated by the light source due to the reflection of the light source box wall. We tested the light irradiated intensity at multiple positions of the device and found that the light intensity at the top position was 1 kW m^{-2}, the intensity at both sides was 0.94 kW m^{-2}, and the light intensity at the bottom position was 0.91 kW m^{-2}. It indicated that all the surface of the device can be irradiated by light. Therefore, we assumed that all Bi_2Te_3/Cu layer could be irradiated by light. The diameter of the reaction tube was 3 cm (D), and its length was 50 cm (L). According to the formula of calculated

cylindrical side area S = D*L*π (3cm*50cm*3.14) = 0.0471 m^2, which was recognized as irradiation area.
