# Supplementary material for: General heterostructure strategy of photothermal materials for scalable solar-heating hydrogen production without the consumption of artificial energy
Source: Nat Commun. 2022 Feb 9;13:776. doi: 10.1038/s41467-022-28364-y (PMC8828830; doi:10.1038/s41467-022-28364-y)
Supplement: Supplementary file 3 — Description of Additional Supplementary Files [file 41467_2022_28364_MOESM3_ESM.pdf]

Description of Additional Supplementary Files

File Name: Supplementary Movie 1
Description: The panoramic video of outdoor solar-heating system used for hydrogen production from MSR.
